# Effect of tendon vibration during wide-pulse neuromuscular electrical stimulation (NMES) on muscle force production in people with spinal cord injury (SCI)

**DOI:** 10.1186/s12883-018-1020-9

**Published:** 2018-02-13

**Authors:** Vanesa Bochkezanian, Robert U. Newton, Gabriel S. Trajano, Amilton Vieira, Timothy S. Pulverenti, Anthony J. Blazevich

**Affiliations:** 1 Department of Exercise and Health Sciences, School of Health, Medical and Applied Sciences, Central Queensland University, Building 34.1.02, Bruce Highway, North Rockhampton, Qld 4702 Australia; 2 Exercise Medicine Research Clinic, Edith Cowan University, Perth, Australia; 3 Centre for Sports and Exercise Science, School of Medical and Health Sciences, Edith Cowan University, Joondalup, Australia; 4 UQ Centre for Clinical Research, The University of Queensland, Brisbane, Australia; 5 School of Exercise and Nutrition Sciences, Institute of Health and Biomedical Innovation, Queensland University of Technology (QUT), Brisbane, Australia; 6 UDF-University Centre, Brasilia, Brazil

## Abstract

**Background:**

Neuromuscular electrical stimulation (NMES) is commonly used in skeletal muscles in people with spinal cord injury (SCI) with the aim of increasing muscle recruitment and thus muscle force production. NMES has been conventionally used in clinical practice as functional electrical stimulation (FES), using low levels of evoked force that cannot optimally stimulate muscular strength and mass improvements, and thus trigger musculoskeletal changes in paralysed muscles. The use of high intensity intermittent NMES training using wide-pulse width and moderate-intensity as a strength training tool could be a promising method to increase muscle force production in people with SCI. However, this type of protocol has not been clinically adopted because it may generate rapid muscle fatigue and thus prevent the performance of repeated high-intensity muscular contractions in paralysed muscles. Moreover, superimposing patellar tendon vibration onto the wide-pulse width NMES has been shown to elicit further increases in impulse or, at least, reduce the rate of fatigue in repeated contractions in able-bodied populations, but there is a lack of evidence to support this argument in people with SCI.

**Methods:**

Nine people with SCI received two NMES protocols with and without superimposing patellar tendon vibration on different days (i.e. STIM and STIM+vib), which consisted of repeated 30 Hz trains of 58 wide-pulse width (1000 μs) symmetric biphasic pulses (0.033-s inter-pulse interval; 2 s stimulation train; 2-s inter-train interval) being delivered to the dominant quadriceps femoris. Starting torque was 20% of maximal doublet-twitch torque and stimulations continued until torque declined to 50% of the starting torque. Total knee extensor impulse was calculated as the primary outcome variable.

**Results:**

Total knee extensor impulse increased in four subjects when patellar tendon vibration was imposed (59.2 ± 15.8%) but decreased in five subjects (− 31.3 ± 25.7%). However, there were no statistically significant differences between these sub-groups or between conditions when the data were pooled.

**Conclusions:**

Based on the present results there is insufficient evidence to conclude that patellar tendon vibration provides a clear benefit to muscle force production or delays muscle fatigue during wide-pulse width, moderate-intensity NMES in people with SCI.

**Trial registration:**

ACTRN12618000022268. Date: 11/01/2018. Retrospectively registered.

## Background

Spinal cord injury (SCI) is most commonly caused by trauma and interrupts the connection between supra-spinal and spinal regions of the CNS [1]. This leads to a reduction of the voluntary activation of muscles below the lesion level, reducing muscle force production, impairing physical function, and profoundly compromising physical health and quality of life (QoL) [1–3]. Furthermore, the reduction in muscle force production is a major predictor of mortality risk and seems to be partly attributable to the quantity (i.e. absolute muscle volume) and quality (i.e. muscle density) of muscle mass [4, 5].

A common method to increase muscular force production and muscle mass is the use of neuromuscular strength training. Particularly, high-intensity muscle contractions have been proven to enhance longevity and QoL in different clinical populations, such as diabetes type 2 and osteoporosis, as well as in older adults [6–9]. However, muscle strength training poses an increased challenge for people with a neurological condition, such as people with SCI. Alternatively, neuromuscular electrical stimulation (NMES) is a commonly used intervention in rehabilitation programs to increase muscle recruitment and thus muscle force production, especially in individuals with a complete loss of motor function [10–12]. NMES has been conventionally used in clinical practice as functional electrical stimulation (FES), i.e. a prolonged and low levels of evoked force NMES exercise paired simultaneously or intermittently with a functional task [12]. However, such interventions cannot optimally stimulate muscular strength and mass improvements, imposing a higher load to the muscle to obtain higher force output [13, 14] in accordance with the overload training principle [15], to obtain musculoskeletal changes in paralysed muscles.

The use of high intensity intermittent NMES training (hereafter referred to as near-maximal evoked muscle contractions) as a strength training tool has not been adopted clinically in people with SCI, probably due to the lack of evidence from long-term intervention trials documenting its physiological effects [16, 17] but mainly because these types of protocols generate rapid muscle fatigue and thus prevent the performance of repeated high-intensity muscular contractions [18–21]. This muscle fatigue is exacerbated in people with SCI due to the loss of fatigue-resistant motor units and their higher proportion of more fatigable motor units [22–26], making the rapid muscle fatigue induced by NMES a continuing issue [27, 28]. A clinical issue is therefore presented since higher volumes of strength training, in comparison to lower volumes, are known to promote greater health and functional adaptations, particularly in older adults and in clinical populations such as people with diabetes type 2 [29, 30]. For these reasons, early muscle fatigue represents a problem and, if suboptimal stimulation parameters are used and NMES training elicits a rapid muscle fatigue, may prevent the delivery of the ideal dose of training to realise optimum musculoskeletal adaptations in paralysed muscles. Nonetheless, NMES as a strength training modality has previously been shown to stimulate quadriceps muscle hypertrophy [31–34], improved skeletal muscle oxidative capacity [35] and to improve lean muscle mass [33] and bone adaptations in people with SCI [36–38]. Yet, the use of NMES as high-intensity strength training mode has not been extensively investigated and, due to the limited evidence supporting its use for increasing muscle strength [39, 40] is not commonly used in clinical practice.

One promising method to enhance force production whilst minimising muscle fatigue is the application of tendon vibration [41, 42]. Tendon vibration may generate trains of Ia-afferent signals to the spinal cord that induce a progressive excitation of homonymous motor neurones and promote the development of persistent inward calcium (Ca^2+^) or sodium (Na^+^) currents at their dendritic trees [43–45]. Thus, this method could amplify and prolong the synaptic input and create a sustained depolarisation leading to an increased recruitment of motor units (i.e. self-sustained firing) and increase muscle force production, especially when coupled with wide-pulse width (e.g. 1000 μs) NMES [46, 47]. Importantly, however, vibration tends to preferentially excite low-threshold (i.e. fatigue resistant) motor units [48], which exhibit significant endurance and are therefore likely to contribute to a great total muscular work before fatigue is induced. In previous studies the positive effects of vibration were observed in able-bodied populations during wide-pulse width NMES applied both at relatively low (~ 5% MVC force) [46] and high (~ 20% MVC force) [47] levels of muscle force production, suggesting that vibration may augment muscle contraction force and allow a greater total muscular effort before fatigue during wide-pulse width NMES even when relatively high contraction intensities are used, such as those required for strength training. However, it is unknown whether the imposition of tendon vibration onto wide-pulse width NMES promotes such benefits in people with SCI. In fact, it is also not known whether tendon vibration might elicit an additional excitation of the motor neuron pool through the same (central) pathways as wide-pulse width NMES [43–45, 49] and therefore instead promote a greater muscle fatigue in partially or completely paralysed muscles.

In a previous study [50], patellar tendon vibration during wide-pulse width NMES in able-bodied individuals resulted in an increased total muscle work performed in “positive responders” only, but a reduced total work in others. Nonetheless, within the whole study cohort the application of patellar tendon vibration tended to minimise voluntary muscle fatigue caused by the NMES [50], and would thus have allowed the study participants to immediately perform other physical activities without a notable negative impact on performance. Thus, it is of great interest to determine whether similar effects would be obtained in people with SCI. The purpose of the present study, therefore, was to determine whether patellar tendon vibration superimposed onto wide-pulse width NMES under standard clinical conditions elicits a greater peak muscle force with less muscle fatigue (i.e. a greater total impulse) when compared to NMES applied without patellar tendon vibration in people with SCI. We hypothesised that patellar tendon vibration superimposed onto wide-pulse width NMES would elicit a greater peak muscle force with less muscle fatigue (i.e. a greater total impulse) that NMES applied without patellar tendon vibration in people with SCI.

## Methods

### Subjects

Nine subjects with SCI (3 females, 6 males) were recruited from the Spinal Cord Injuries Australia (SCIA) Activity-based therapy exercise program “NeuroMoves” and the Perth community (mean ± SD, age: 39.4 ± 10.6 y; height: 176.2 ± 9.7 cm; body mass 80.6 ± 9.6 kg). Four subjects were classified as complete spinal cord injury (AIS A) and five were classified as incomplete (AIS B-D). All subjects were recruited by word of mouth or email and had a SCI of more than 6 months. Prior to the study, subjects were given detailed information about the procedures and the risks of participating in the study, and they all signed a written consent form. Subjects completed the Physical Activity Readiness Questionnaire (PAR-Q) to ensure safe exercise participation and refrained from vigorous exercise (48 h) and alcohol (24 h) and stimulant consumption (e.g. caffeine, energy drinks for 6 h) prior to testing. Participants included in this study had medical clearance and were fit and medically stable to participate in this study. Participants were asked to replicate the same physical routine for each session. Sessions were completed at the same time of the day and under the same experimental conditions. This research study was approved by the Edith Cowan University Human Ethics Committee (reference number: 11,623). Subject characteristics are detailed in Table 1.

**Table 1:** Subject characteristics. Subject levels of injury, completeness of lesion, time since injury, AIS scale score, medication type and response to tendon vibration (i.e. whether a tendon vibration reflex response is detectable). Complete (C) lesion means no voluntary muscle contraction below the level of injury. Incomplete (I) lesion means some voluntary muscle contraction below the level of injury [71]

Subject	Level of injury	Complete (C)/Incomplete (I)	Time since injury (y)	AIS	Medication	Positive responder to tendon vibration	Negative responder to tendon vibration
A	T_7_	I	3	B	Baclofen		x
B	T_6_	C	6	A	Oxybutin		x
C	T_6_	C	5	A	Baclofen		x
D	C_6_-C_7_	C	4	A	Baclofen		x
E	T_12_	I	2	D	Baclofen		x
F	C_7_	C	1	A	Baclofen	x	
G	T_5_	I	20	D	Baclofen	x	
H	T_3_	I	2	B	Baclofen	x	
I	L_3_	I	4	D	N/A	x	

### Procedures

The procedures used in this study followed the methodology described in a previous study in healthy individuals [50], but was adapted to people with SCI accordingly. All subjects attended the Neuromuscular Physiology Laboratory at Edith Cowan University on three occasions on different days (1 day per week for the duration of 4 weeks) with a minimum of 7 days between sessions. One week before starting data collection, the subjects attended a full familiarisation session where the NMES protocol was applied to the dominant quadriceps femoris with and without simultaneous patellar tendon vibration. Each subject received 1 min of tendon vibration and 3 min of NMES to ensure they could tolerate the intervention; all subjects tolerated the NMES and tendon vibration protocols well. The subsequent two sessions were used to complete the following two experimental protocols in a random order without replication: 1) NMES only (STIM); and 2) NMES superimposed onto tendon vibration (STIM+Vib).

In familiarisation and experimental sessions, the subjects were asked to produce a voluntary knee extension contraction of approximately 50% of perceived maximal voluntary exertion while seated on an isokinetic dynamometer***.*** If any voluntary contractions were visualised and recorded, then a standardised warm-up protocol was performed, consisting of isometric knee extensions at 30%, 50%, 70% and 90% of perceived maximal effort before performing a series of three knee extension MVICs. However, if no voluntary contraction was recorded then three attempts of maximal voluntary contractions (MVICs) were instructed without warm-up efforts. The subjects were seated with hip and knee joint angles of 85^o^ and 90^o^, respectively (0 ^o^ = full knee extension), with the thigh and trunk secured to the dynamometer chair and the knee joint aligned with the center of rotation of the dynamometer. All subjects were instructed to produce a force against the dynamometer arm by extending the knee as fast and hard as possible for 3 s, and verbal encouragement and visual feedback were provided during all MVICs irrespective of the subject’s ability to voluntarily activate their lower limbs. This method was implemented to be consistent with the procedures among all subjects.

### Electrical stimulation and tendon vibration protocols

NMES was delivered by a high-voltage constant-current electrical stimulator (400 V, DS7A, Digitimer Ltd., Welwyn Garden City, UK) through four self-adhesive stimulation electrodes (Axelgaard, PALS, USA) placed over the rectus femoris (RF), vastus lateralis (VL), and vastus medialis (VM); two 5 × 10 cm electrodes were placed over RF and one 5 × 5 cm electrode was placed on each of the VM and VL, approximately at their motor points. The electrodes were then moved if necessary to elicit the greatest twitch response with low stimulation intensity in every session [51]. The electrode positions were marked on a plastic sheet for each subject and indelible ink was used to mark these positions on the skin to ensure identical electrode placement at subsequent sessions.

To habituate the subjects to the electrical stimulations- and after the attempts of MVCIs, two electrical square-wave stimuli (1000 μs square-wave pulses separated by 5 ms) were delivered to the dominant leg (determined in familiarisation session using NMES) every 20 s while the stimulation current was increased from 30 to 99 mA in 10-mA increments until a plateau in the maximum peak twitch torque was observed. This was defined as maximal peak twitch torque (τ_tw,p_) and was used as the “target torque”. A second, submaximal peak twitch torque (τ_tw,sub_) recording was obtained at 40 mA and retained for analysis of changes in submaximal torque, to assess the ability of the muscle to contract under submaximal conditions, which are often used in a clinical context. Subsequently, a maximum of three trains of NMES (described below) were performed at different stimulation current intensities until reaching the closest value to the target torque. This procedure was replicated in all participants regardless of their response to the imposed current. All participants in this study showed an increase in evoked torque when increasing the current from 30 to 99 mA (maximum output allowed by the electrical stimulator) and the level of evoked torque was set equal or close to the maximal peak twitch torque (τ_tw,p_).

The NMES protocol consisted of repeated 30-Hz trains of 58 wide-pulse width (1000 μs) symmetric biphasic pulses (0.033-s inter-pulse interval), where a single train duration was 2 s and the inter-train interval was 2 s (i.e. 2-s on and 2-s off). The level of evoked torque was set equal or close to the maximal peak twitch torque (τ_tw,p_), which should be equivalent to ~ 20% of MVIC.

Patellar tendon vibration was applied using a vibration device (Deep Muscle Stimulator, Las Vegas, NV, USA), which mechanically vibrated the tendon at 55 Ha and amplitude of 7 mm (confirmed through direct measurement of high-speed video). The two test conditions were:

- **STIM**: Electrically-evoked muscle contractions produced by delivering the NMES protocol until torque was reduced to ≤50% of the target torque (i.e. τ_tw,p_) in one electrically-evoked contraction, which was defined as “target fatigue”.
- **STIM + Vib**: Electrically-evoked contractions delivered as in STIM, but superimposed with patellar tendon vibration which was applied for at least 5 s before NMES and after target fatigue was reached.

**Fig. 1:**
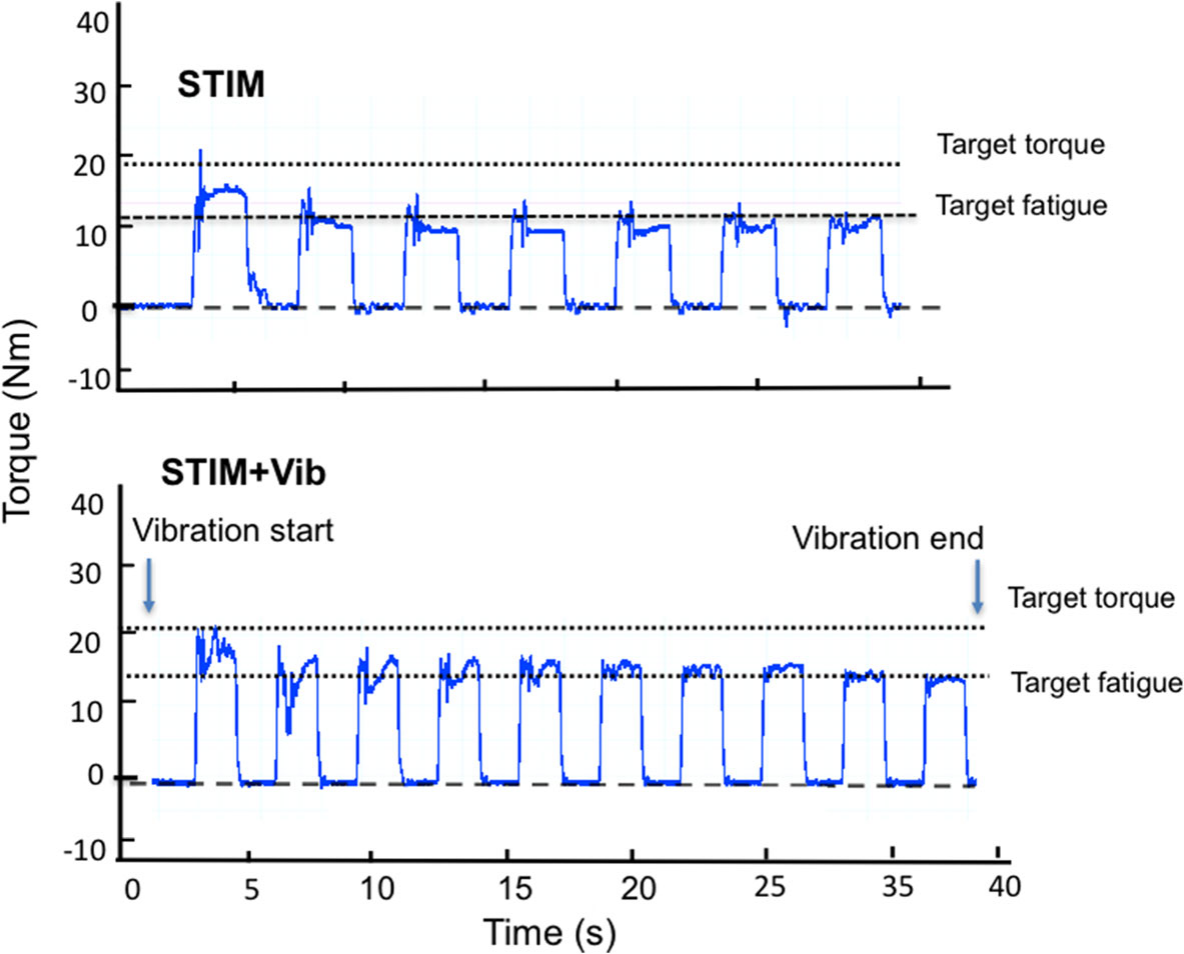
Graphical representation of the STIM and STIM+Vib. STIM and STIM+Vib protocols in one of the participants can be observed in this graph. Blue traces refer to the torque forces evoked by the NMES protocols

For a graphical representation of the STIM and STIM+Vib protocols please refer to Fig. 1.

### Data collection and analysis

#### Peak evoked torque, torque-time integral and number of contractions

Two measures of peak-twitch evoked torque were analysed: one submaximal (τ_tw,sub_) evoked at a 40-mA current, and one maximal (τ_tw,p_) obtained from the peak of the torque-current relationship. These were obtained both before and after the NMES protocols were delivered. The total torque-time integral (TTI) was used to provide a measure of the total exercise stimulus received by the muscle in each condition. TTI was calculated as the product of torque and time calculated from the onset of the first stimulation train (STIM) or vibration onset (STIM+Vib) to the end of the final evoked contraction when “target fatigue” was reached (as defined in Procedures). Peak evoked torque was defined as the highest torque value obtained after the onset of the first stimulation train for both STIM and STIM+Vib. TTI and peak evoked torque were compared between STIM and STIM+Vib. The post-study data analysis revealed that some subjects responded with a greater TTI after STIM+Vib than STIM (i.e. positive responders to patellar tendon vibration) whilst others showed no difference or a lower TTI after STIM+Vib than STIM (i.e. negative responders to patellar tendon vibration), as described in Results. Therefore, a second analysis was performed after separating subjects into positive and negative responders to patellar tendon vibration (described in Procedures). Positive and negative responders were identified not only during the training sessions but also during pilot testing sessions where different NMES protocols with superimposed patellar tendon vibration were utilised. Although the pilot data are not presented in the present article, this information reinforced the identification of positive and negative responders to patellar tendon vibration during at least one pilot session and provided confidence in the interpretation of results. The total number of contractions was measured as the number of contractions from the beginning of the first evoked contraction reaching the target torque until the last contraction when reaching 50% of the target torque (“target fatigue”).

### Statistical analysis

Two-way repeated measures analysis of variance (ANOVA) was used to compare changes in all variables between conditions (STIM, STIM+Vib) over time (PRE, POST). A Wilcoxon test was conducted to compare STIM, STIM+Vib between PRE-and POST in positive and negative responders to patellar tendon vibration whilst using assessments at PRE as the covariates. Pairwise t-tests were performed when significant interaction effects were found. A chi- square test for independence was used to assess whether an association existed between subjects with complete and incomplete SCI and the likelihood of being a positive or negative responders. Statistical significance was set at an alpha level of *p* ≤ 0.05 and values are reported as mean ± SD.

## Results

### Peak evoked torque, torque-time integral (TTI) and total number of contractions

**Fig. 2:**
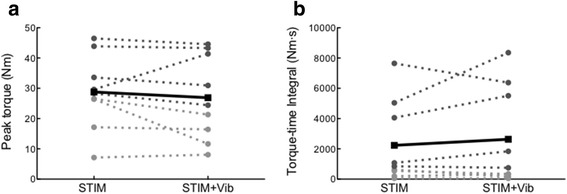
Peak torque and Torque-Time Integral (TTI) in STIM and STIM+Vib conditions. **a** Peak torque production (Nm) in STIM and STIM+Vib conditions for all subjects. No statistically significant differences were found between the two conditions **b** Torque-time integral (TTI; Nm⋅s) in STIM and STIM+Vib conditions for all subjects_._ No statistically significant differences were found between the two conditions_._ Grey dashed lines represent individual subjects and the black solid line represents the group mean. Darker grey dots indicate subjects with incomplete spinal cord injury (SCI) and light grey dots indicate subjects with complete SCI

**Fig. 3:**
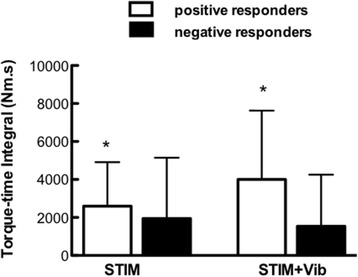
Torque-Time Integral (TTI; Nm⋅s) in STIM and STIM+Vib. TTI recorded in STIM and STIM+Vib conditions for positive and negative responders to tendon vibration. Significant increases of 59.2 ± 15.8% in TTI in STIM+Vib compared to STIM for positive responders to tendon vibration (*p* = 0.13) and decreases of − 31.3 ± 25.7% in STIM+Vib for negative responders to tendon vibration (*p* = 0.14) were observed, when using τ_tw,p_ at PRE for both conditions as a covariate (*p* = 0.02), however TTI was not statistically different between conditions. * Significantly different between positive and negative responders (*p* < 0.05)

No statistical differences in peak evoked torque (*p* = 0.43; Fig. 2a), torque-time integral (TTI; *p* = 0.39; Fig. 2b) or total number of contractions (*p* = 0.78) were observed between STIM and STIM+Vib. Nonetheless, the response to STIM+Vib (based on TTI) was clearly greater than in STIM in some subjects (40% of sample) but lesser (or negative) in others. Thus, an additional comparative analysis of positive versus negative responders to patellar tendon vibration was undertaken, where positive responders to patellar tendon vibration were defined as those subjects who responded with a greater TTI in STIM+Vib when compared to STIM. This analysis revealed no statistical difference in TTI between STIM and STIM+Vib for positive or negative responders (Fig. 3), or a difference between STIM and STIM+Vib for the whole cohort collectively. However, the between-condition difference was dissimilar between the responder groups when using τ_tw,p_ at PRE for both conditions as a covariate (*p* = 0.02); TTI was 59.2 ± 15.8% greater in STIM+Vib than STIM in positive responders to patellar tendon vibration (*p* = 0.13) but 31.3 ± 25.7% less in STIM+Vib than STIM in negative responders (*p* = 0.14), as shown in Fig. 3. There was also no clear effect of completeness of lesion (complete or incomplete SCI) on the likelihood of being a positive or negative responder to tendon vibration (χ^2^ (1, *n* = 9) = 1.10, *p* = 0.70). Also, no significant differences were found between the conditions (STIM and STIM+Vib) for total number of contractions, despite a trend being observed; the mean total number of contractions for positive responders to tendon vibration for STIM was 56.5 ± 60.5 and for STIM+Vib was 70.2 ± 68.9, whilst the means for negative responders to tendon vibration were 41.6 ± 37.4 for STIM and 33.8 ± 30.9 for STIM+Vib (figure not shown).

### Muscle force measures: Maximal evoked force (τ_tw,p_) and submaximal evoked force (τ_tw,sub_)

**Fig. 4:**
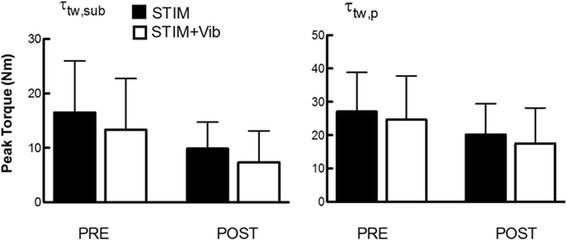
Submaximal (_τtw,sub)_ and maximal_(τtw,p)_ peak twitch torque recorded before (PRE) and after (POST) STIM and STIM+Vib. Submaximal (_τtw,sub: 40 mA) and maximal (τtw,p) electrical stimulation peak twitch torques recorded before (PRE) and after (POST) STIM and STIM + Vib._ Submaximal twitch torque (τ_tw,sub; top panel_) declined 40.4 ± 4.7% and maximal force (τ_tw,p; bottom panel_) declined 27.0 ± 5.0% of baseline in STIM, whilst τ_tw,sub_ declined 45.0 ± 4.2% and τ_tw,p_ declined 30.6 ± 5.0% of baseline in STIM+Vib. However, _no statistically significant differences were found between the two conditions_

There was a significant effect of time, as the relative maximal force (τ_tw,p_; *p* = 0.00) and submaximal force (τ_tw,sub_; *p* = 0.00) decreased from PRE to POST in both conditions (STIM and STIM+Vib). However, a similar pattern was observed in STIM+Vib and STIM conditions, as there were no significant effects of condition (τ_tw,p_: *p* = 0.21; τ_tw,sub_: *p* = 0.13) and no significant condition × time interaction (τ_tw,p_: *p* = 0.98; τ_tw,sub_: *p* = 0.77). Submaximal twitch torque (τ_tw,sub_) declined by 40.4 ± 4.7% and maximal force (τ_tw,p_) declined by 27.0 ± 5.0% of baseline in STIM, whilst τ_tw,sub_ declined by 45.0 ± 4.2% and τ_tw,p_ declined by 30.6 ± 5.0% of baseline on STIM+Vib (Fig. 4).

## Discussion

The purpose of this study was to determine whether patellar tendon vibration superimposed onto wide-pulse width NMES would elicit greater peak muscle force production and/or induce less muscle fatigue (i.e. increase the total impulse before fatigue) than NMES alone in people with spinal cord injury (SCI). The main finding was that the torque-time integral (TTI) measured at the point of fatigue (i.e. 50% of initial evoked torque) was not statistically different between STIM and STIM+Vib conditions, although a significantly greater TTI was found in four (of nine) “positive” responders to patellar tendon vibration (59.2 ± 15.8%). Thus, in four of the present participants, the addition of the patellar tendon vibration allowed for a greater total muscular work to be performed, but a lower total muscular work was completed in the other five subjects (− 31.3 ± 25.7%). However, these changes were not statistically different between conditions. Moreover, as observed from Fig. 2, there was no evidence that completeness of lesion (complete vs. incomplete SCI) was a factor influencing the likelihood of being a responder to patellar tendon vibration. Therefore, whilst the imposition of patellar tendon vibration during wide-pulse width NMES may allow for a greater impulse to be provided before fatigue in some individuals, it is not apparently clear who might benefit from the application of patellar tendon vibration in people with SCI based on our results.

The use of patellar tendon vibration superimposed onto wide-pulse width NMES was hypothesised to allow for a greater training impulse prior to fatigue, which might be of practical significance since it might evoke greater chronic increases in muscle strength and mass as well as improve muscle performance after a period of training [36, 52]. However, this appears to be the case only in some individuals who show a positive response to patellar tendon vibration. The ability to produce greater forces after a period of training may allow for higher volumes of muscle work to be performed during a physical rehabilitation session and thus generate early improvements in physical performance and physical health benefits in people with SCI; this hypothesis should thus be tested using a longitudinal study design. A reflex response induced by patellar tendon vibration may facilitate the initiation, maintenance and strength of any residual voluntary contraction in people with incomplete SCI. The use of patellar tendon vibration in those with a complete lesion can help to regulate the reflex response, which is highly altered after a complete SCI, by stimulating reflex pathways. Previous research has shown that somatosensory information continues to flow in a modified manner after a complete SCI and that there is a potential to generate motor patterns if this somatosensory input is stimulated by the use of methods such as NMES and tendon vibration [53, 54]. The mechanisms of action of tendon vibration relate to the tonic vibration reflex (TVR), elicited by the application of tendon vibration, which may potentially help recruit more motor units and thus increase muscle force production when used in combination with NMES [46]. Greater muscular forces elicited by the tendon vibration when superimposed to NMES were found in previous studies in able-bodied people [46] and were attributed to the generation of persistent inward currents, recruiting higher-threshold motor units through reflexive pathways and increasing muscle force production [55, 56]. On the other hand, the reduced training impulse prior to fatigue observed in the negative responders to patellar tendon vibration suggested that patellar tendon vibration may be detrimental for some people and that in these people the use of (wide-pulse width) NMES alone may be more beneficial. This response observed in negative responders to patellar tendon vibration may speculatively be caused by the low-to-moderate frequency (55 Hz) vibration resulting in a stimulation of Golgi tendon organs, which can cause autogenic inhibition and potentially decrease the motor output [57]. Therefore, the present, individual-specific results cannot confirm the hypothesis that superimposing patellar tendon vibration onto wide-pulse width NMES would elicit an additional greater peak muscle force with less muscle fatigue (i.e. a greater total impulse) when compared to NMES applied without patellar tendon vibration in most people with a SCI.

These variable responses to patellar tendon vibration may also be explained by the highly individual functional deficits found between individuals with incomplete SCI, where the transmission of the sensory-motor information is altered at the synaptic level [58, 59] and there is a muscle spindle afferent dysfunction in both complete and incomplete lesions to the spinal cord [58, 59]. A similar inter-individual variability in response to wide-pulse width NMES has also been suggested to originate from a difference in monoamine levels between individuals [43] and could be one reason for the different response to patellar tendon vibration superimposed onto wide-pulse width NMES observed in the current study. This difference in monoamine levels could be exacerbated by the altered neuromuscular system in people with spinal cord injuries [60], such as changes in the excitability of the motor neurone [61], fibre type transformation towards fast-fatigable [60] and high levels of muscle atrophy [62]. Another factor that could have decreased the stretch reflex and thus prevented the formation of PICs is the use of antispasmodic medications, such as Baclofen [63]. On the other hand, the increased TTI in positive responders to patellar tendon vibration might be attributable to the development of tonic vibration reflexes (TVR) which increase muscle force contributions between the evoked muscular contractions [47, 64]. However, the activation of already hyper-excitable sensory pathways by the use of patellar tendon vibration in people suffering from a spinal cord injury may have either triggered episodes of intrinsic phasic spasticity in some participants [65], whilst attenuated spasticity symptoms in others [66], and thus may have increased the variability in the response to wide-pulse width NMES and patellar tendon vibration observed in the present study. These hypotheses will need to be explored in further studies investigating explicitly the pathophysiological responses (i.e. spasticity) of paralysed muscles to tendon vibration superimposed onto wide-pulse width NMES.

Another important finding of the current study was that the decline in evoked muscle force was not attenuated by the application of patellar tendon vibration. Muscle fatigue experienced after both STIM and STIM+Vib could be attributed to the “peripheral fatigue” (i.e. contractile alterations) induced by NMES, due to the repeated activation of the same muscle fibres, especially due to the missing sensory feedback from the muscles to the spinal cord to prevent failure after a SCI [67–69]. It may also be possible that patellar tendon vibration activated not only excitatory but also inhibitory interneurons and thus negated the possible positive effects of patellar tendon vibration on muscle fatigue [70]. Accordingly, results from this study are inconclusive regarding the effects of superimposing patellar tendon vibration onto wide-pulse width NMES in people with SCI and future research studies are needed to investigate the mechanisms of action of tendon vibration in chronically paralysed muscles. Moreover, the present results remain to be verified in future studies in a larger cohort of people with SCI, whilst also considering the potentially-confounding factors of age, level of injury, AIS classification (complete vs. incomplete), time since injury, spasticity and medication use, which may impact in the measured outcomes.

## Conclusion

The imposition of patellar tendon vibration onto moderate-frequency, wide-pulse width NMES may allow for a greater amount of muscular work (muscle force production) to be completed before fatigue in a proportion of participants (i.e. positive responders to tendon vibration) with SCI. However, a lesser response may also be elicited in participants who respond negatively to patellar tendon vibration (60% in the current study) and thus, for those cases, wide-pulse width NMES alone may provide greater benefits. The use of patellar tendon vibration superimposed onto wide-pulse width NMES did not minimise the (peripheral) fatigue elicited by the training session. Based on the present results there is insufficient evidence to conclude that patellar tendon vibration improves muscle force production during the use of wide-pulse width NMES in people with SCI. In the clinic, it is suggested that people with SCI are assessed for their response to patellar tendon vibration in order that the appropriate protocol is implemented. Nonetheless, replication of these findings is required before clear decisions as to whether to implement the strategy in clinical practice can be made.

## Abbreviations

τ_tw,p_: Maximal evoked torque (peak twitch torque)
τ_tw,sub_: Submaximal evoked torque (peak twitch torque)
AIS: American Spinal Injury Association Impairment Scale
FES: Functional electrical stimulation
MVC: Maximal voluntary contraction
MVIC: Maximal voluntary isometric contraction
NMES: Neuromuscular electrical stimulation
QoL: Quality of life
RF: Rectus femoris
SCI: Spinal cord injury
STIM: Neuromuscular electrical stimulation protocol
STIM+Vib: Neuromuscular electrical stimulation superimposed onto tendon vibration protocol
TTI: Torque-time integral
VL: Vastus lateralis
VM: Vastus medialis
